# Enhancing adipose tissue functionality in obesity: senotherapeutics, autophagy and cellular senescence as a target

**DOI:** 10.1186/s40659-024-00531-z

**Published:** 2024-08-08

**Authors:** Consuelo Arias, Javiera Álvarez-Indo, Mariana Cifuentes, Eugenia Morselli, Bredford Kerr, Patricia V. Burgos

**Affiliations:** 1 Escuela de Kinesiología, Facultad de Odontología y Ciencias de la Rehabilitación, Universidad San Sebastián, Santiago, 7500922 Chile; 2 Centro de Biología Celular y Biomedicina (CEBICEM), Facultad de Medicina y Ciencia, Universidad San Sebastián, Santiago, Chile; 3 Institute of Nutrition and Food Technology (INTA), University of Chile, Santiago, Chile; 4 Advanced Center for Chronic Diseases (ACCDiS), Santiago, Chile; 5 Department of Basic Sciences, Faculty of Medicine and Sciences, Universidad San Sebastián, Santiago, Chile; 6 Centro Basal Ciencia & Vida, Universidad San Sebastián, Santiago, Chile

## Abstract

Obesity, a global health crisis, disrupts multiple systemic processes, contributing to a cascade of metabolic dysfunctions by promoting the pathological expansion of visceral adipose tissue (VAT). This expansion is characterized by impaired differentiation of pre-adipocytes and an increase in senescent cells, leading to a pro-inflammatory state and exacerbated oxidative stress. Particularly, the senescence-associated secretory phenotype (SASP) and adipose tissue hypoxia further impair cellular function, promoting chronic disease development. This review delves into the potential of autophagy modulation and the therapeutic application of senolytics and senomorphics as novel strategies to mitigate adipose tissue senescence. By exploring the intricate mechanisms underlying adipocyte dysfunction and the emerging role of natural compounds in senescence modulation, we underscore the promising horizon of senotherapeutics in restoring adipose health. This approach not only offers a pathway to combat the metabolic complications of obesity, but also opens new avenues for enhancing life quality and managing the global burden of obesity-related conditions. Our analysis aims to bridge the gap between current scientific progress and clinical application, offering new perspectives on preventing and treating obesity-induced adipose dysfunction.

## Introduction

Obesity is a complex and multi-systemic chronic disease that affects multiple systemic processes impacting on the maintenance of corporal energy homeostasis. Nowadays, obesity is considered as a global pandemic and the World Health Organization (WHO) reports that the rate of obesity has nearly tripled since 1975, currently affecting over one billion individuals globally [1, 2]. Adipose tissue, which used to be considered as a mere reservoir of fat, is now recognized as an endocrine organ with important functions in the regulation of metabolism and homeostasis. Its dysfunction significantly impacts overall health and is considered a key risk factor for many common diseases [3]. The excessive accumulation of fat disrupts corporal energy balance and metabolic homeostasis, leading to systemic inflammation, dyslipidemia (abnormal blood lipids levels), and insulin resistance, which have been linked to the development of various chronic non-communicable pathologies, such as cardiovascular disease, type 2 diabetes, and even certain types of cancers such as esophagus, gastric, colon and postmenopausal breast cancer [4–7]. These diseases impose a significant economic burden [4] and the World Obesity Atlas 2023 mentions that by 2035 nearly 3% of global gross domestic product will be destined to the economic impact of overweight and obesity [5].

## Physiology of adipose tissue

The human body contains various types of adipose tissue, each with a unique profile of bioactive peptides or proteins known as adipokines, a type of cytokines specifically secreted by adipocytes with important roles in metabolism [6–8]. These tissues include white adipose tissue (WAT), that mainly serves as a reservoir for fat storage that is further categorized into subcutaneous (SAT) and visceral depots (VAT), which is considered ectopic fat when it is located for example in liver and muscle. Additionally, brown (BAT) and beige adipose tissue have been described, mainly with a thermogenic rather than a lipid-storing role in the organism [2, 9, 10].

In general, healthy WAT is essential for maintaining energy balance by storing excess energy as triglycerides, thereby preventing hyperlipidemia and ectopic fat formation. Energy is stored intracellularly within lipid droplets, an organelle that is a hallmark of differentiated adipocytes and functional adipose tissue [11]. Crucially, during periods of energy deficit, adipocytes hydrolyze these triglycerides to release fatty acids, serving as an effective buffering system between energy storage and release. WAT contains various cell types, including mature adipocytes, preadipocytes, fibroblasts, endothelial cells, and immune cells, where the proportions of these cellular subtypes can vary based on metabolic conditions and aging [12, 13]. Furthermore, WAT serves as a robust source of adipose progenitor cells (APC). These multipotent stromal cells possess the capability to differentiate into preadipocytes. Upon further differentiation, these cells undergo a process termed adipogenesis [14].

Beyond its function as an energy storage deposit, adipose tissue also exhibits endocrine functionality, secreting a range of adipokines [15, 16], instrumental in modulating the metabolic activities of diverse tissues, such as muscle, brain, and liver [6]. Adipokines regulate both pathological and physiological processes, and include leptin, adiponectin and resistin, among others [17]. The synthesis of adipokines differs between types of adipose tissue, possibly reflecting a metabolic profile that is particular to each one [8]. Proinflammatory adipokines can directly enhance the generation of many proinflammatory cytokines, such as TNFα, monocyte chemoattractant protein MCP1, and IL-6, favoring the development of metabolic diseases [12]. Other anti-inflammatory adipokines such as adiponectin and omentin play a protective role against obesity co-morbidities and they are downregulated in obesity states [12]. On the other hand, it is known that VAT in individuals with obesity has a higher number of immune cells than SAT, which generates a higher production of proinflammatory cytokines and adipokines reflecting their proinflammatory nature [13]. This phenotypic behavior can change under pathological conditions, as observed in postmenopausal women with obesity, where SAT may also become a major source of circulating proinflammatory adipokines [11, 18]. In individuals with obesity, there is often a diminished secretion of anti-inflammatory and insulin-sensitizing adipokines, such as adiponectin, which are otherwise present at higher levels in lean individuals [18]. Despite the fact that SAT predominantly contains M2 macrophages that are immunomodulatory and instrumental in resolving inflammation, obesity induces a shift, favoring the abundance of pro-inflammatory M1 macrophages, promoting the pro-inflammatory environment characteristic of obesity [19]. Overall, an increase in VAT or SAT in obesity, is associated with an increase in inflammation and several metabolic disorders, such as type 2 diabetes and cardiovascular diseases [2, 6, 8, 20]. Body mass index (BMI), weight and waist circumference (WC) are commonly used as alternative measures to assess obesity in clinical practice due to their convenience and simplicity. However, they do not correlate closely with directly measured fat mass, SAT or VAT, which has been described as a risk factor for cardiovascular disease. Recently, dual-energy X-ray absorptiometry (DXA) has become the preferred method to assess body fat mass, lean mass and bone mineral content in total and specific anatomical regions [21].

During obesity, when caloric intake chronically exceeds energy expenditure, adipose tissue undergoes morphological changes due to both hyperplasia and hypertrophy [17]. Hyperplasia in WAT leads to a significant increase in the proliferation of preadipocytes, which subsequently differentiate into mature adipocytes. In contrast, hypertrophy is characterized by the excessive storage of cytosolic triglycerides in mature adipocytes, causing their increase in size. This hypertrophic condition is associated with elevated expression of inflammatory markers, the release of proinflammatory cytokines and the infiltration of M1-like macrophages, as well as a decrease in insulin signaling. The expansion of adipose tissue is accompanied by a rapid release of fatty acids into the bloodstream upon receiving a lipolytic stimulus, processes which are altered in obesity conditions [11]. Together, these events drive a low-grade systemic chronic inflammation, setting the stage for obesity-related metabolic disorders [2, 9, 15, 22].

## Adipose tissue and obesity: acquisition of the senescent phenotype

**Fig. 1:**
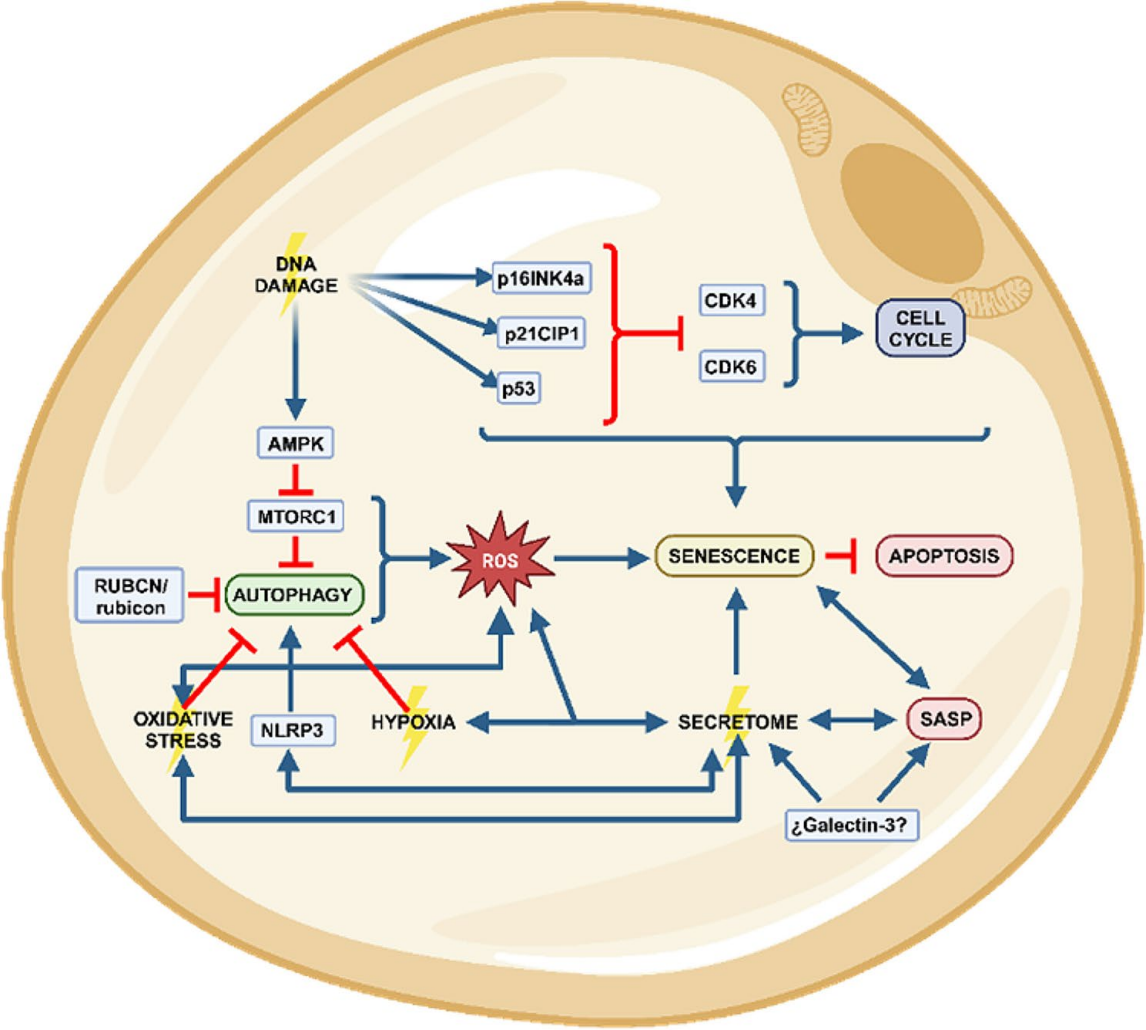
Cellular senescence induction. Cellular senescence can be induced by a variety of stressors. Damaged DNA generates activation of the p53/ p21CIP1 and p16INK4a/RB tumor suppressor pathways, inhibiting cyclin-dependent kinases CDK4, CDK6, which generates deceleration or cessation of the cell cycle and the establishment of a state of perpetual growth arrest. Oxidative stress triggers senescence, autophagy impairment, ROS production and senescent secretome in several cellular contexts. Senescent secretome disrupts autophagy and contributes to SASP. Galectin-3 is a potential molecular link between obesity and the establishment of the senescence phenotype. Hypoxia promotes the production of ROS, disrupts autophagy and stimulates the release of pro-inflammatory adipokines. RUBCN/rubicon inhibits autophagy and promotes senescence. Senescent cells activate different pro-survival pathways to avoid apoptosis. Created with BioRender.com agreement number WZ270NX82C.

Cellular senescence is a multifaceted phenomenon characterized by heterogeneity, particularly accentuated during the aging process [18, 23–25]. In general, senescence is a complex biological process where cells enter a state of permanent growth arrest without undergoing cell death characterized by a cell size enlargement, flattened morphology and enhanced senescence-associated (SA)-β-galactosidase activity [26]. This state can be induced by a variety of stressors such as DNA damage, oncogenic activation, mitochondrial dysfunction, and others [27, 28]. While senescence serves as a crucial mechanism to prevent the proliferation of damaged or potentially cancerous cells, it also contributes to aging and age-related diseases due to the accumulation of senescent cells in tissues. Entry into senescence involves significant changes in cellular physiology, morphology, and gene transcription patterns, leading to functional decline [29]. Gene transcription during cellular senescence requires the activation of specific transcription factors such as retinoblastoma protein (pRB) and tumor protein p53 (p53) [26]. These two proteins are implicated in cell cycle regulation and DNA damage response, that favor the expression of their target genes p16 and p21 (a tumor suppressor genes that inhibits cyclin D–dependent protein kinases), which are involved in cell cycle arrest and used as markers of cellular senescence [26] (Fig. 1). Beyond the activation of p16 and p21, cellular senescence also relies on the continued activation of the mechanistic Target of Rapamycin (mTOR) and Mitogen-activated protein kinases (MAPKs) signaling pathways [30, 31]. These pathways are essential for directly inhibiting the cell cycle progression, thus ensuring that cells remain in a state of arrest [32]. In the context of senescence, mTOR activation can enhance the senescent phenotype by promoting protein synthesis and other metabolic activities that support the maintenance of the senescent state. Similarly, the MAPK signaling pathway, which during senescence is often a response related to stress signals, such as DNA damage or oxidative stress, helps to reinforce the senescent program by promoting the senescence-associated secretory phenotype (SASP) [31, 33] (Fig. 1). SASP comprises the release of proinflammatory, proapoptotic and profibrotic factors [28] that promote the infiltration of immune cells [29, 34]. Several pathways have been described that could regulate the SASP. One of them is the JAK/STAT pathway, which inhibition could alleviates SASP and frailty in old age [35]. Another is the PI3K/Akt/mTOR pathway, where it has been shown that its inhibition in preadipocytes could suppress premature senescence in preadipocytes [36]. This dynamic interplay is crucial for what is known as geroconversion, the process by which cells become hypertrophic (enlarged) and hyperfunctional, a state characterized by an enhanced lysosomal activity and a pronounced secretory phenotype commonly observed during the transition to cellular senescence [29]. Although the stimuli inducing senescence may also activate pathways leading to apoptosis, senescent cells are able to evade this fate by triggering various pro-survival pathways, thereby securing their persistence within tissues, despite their halted proliferation [30]. Adipose tissue has been reported to have one of the highest magnitudes of senescence compared to other tissue types [37]. Moreover, during adipose tissue expansion in obesity, cellular senescence becomes a pivotal event leading to tissue dysfunction [24]. Dysfunctional adipose tissue exhibits a reduction in cellular plasticity, characterized by a poor ability to differentiate into preadipocytes and mature adipocytes, culminating in decreased lipogenesis and increased lipolysis [28]. This process is closely linked to the accumulation of senescent cells not only within adipose tissue but also in various organs, playing a crucial role in the onset and progression of associated chronic diseases [25]. A variety of cells, such as APCs, preadipocytes, and mature adipocytes in WAT, show signs of senescence during obesity [34, 38]– [42]. Senescent APCs and APCs from obese individuals display a diminished proliferative ability affecting their differentiation capacity, showing an upregulation of markers like SA-β-Gal, p53, p21, and p16 [26, 43]– [45]. These markers in APCs can impede their ability to differentiate, particularly into preadipocytes and mature adipocytes, impacting the function of adipose tissue. In fact, committed preadipocytes are decreased in the context of obesity [18], further disrupting the delicate equilibrium between lipid metabolism and lipid storage referred as a disbalance in lipid homeostasis [18]. Moreover, senescence is a self-propagating process that affects surrounding cells that are not in a state of senescence. In this regard, it has been described that primary human senescent fat progenitors secrete activin A, a member of the TGF-β superfamily, which directly inhibits adipogenesis in non-senescent progenitors [46]. Importantly, the senescence phenotype in preadipocytes can be recapitulated in vitro, showing a significant increase in SA-β-Gal activity and markers indicative of cell cycle arrest [47], similar to preadipocytes from aged animals [18, 25]. On the other hand, mature adipocytes, which represent the endpoint of the adipogenic lineage, are also target of senescence [48], similar to the phenotype of aged mature adipocytes which exhibit a notable increase in cell cycle-related proteins such as p53, p21, and p16 [34, 39], which results in a state of perpetual growth arrest [44]. In this regard, Chen et al. reported that aging suppressed adipogenesis and increased expression of SASP factors [49]. This state in mature adipocytes can exacerbate lipid dysregulation, inflammation, and insulin resistance, contributing to the metabolic anomalies often associated with obesity.

## Hypoxia and its role in the development of adipose tissue senescence

Hypoxia plays a pivotal role in the pathological processes of adipose tissue during obesity, including the induction of senescence. Mechanistically, hypoxia leads to the stabilization of hypoxia-inducible factor 1α (HIF-1α), a well-known transcription factor that controls the expression of several genes including those associated with the onset of senescence [50]. Indeed, the stabilization of HIF-1α acts as an early initiator of adipose tissue dysfunction [51]. Notably, HIF-1α affects the metabolism of macrophages within the adipose tissue, promoting the pro-inflammatory M1 macrophage subtype that increases the presence of inflammatory markers in the surrounding secretome [52]. The metabolic change in macrophages directly promotes adipose tissue fibrosis, a pathogenic feature during hypertrophy of adipose tissue [53]. In this regard, subjects with obesity have significantly lower adipose tissue oxygen consumption and blood flow compared to their lean counterparts, resulting from a lower capillary density [53]. Moreover, adipose tissue of individuals with obesity is characterized by a reduction in VEGF levels, a factor involved in angiogenesis, a process needed for the formation of new blood vessels necessary for adequate oxygen supply [54]. Thus, low concentration of VEGF exacerbates the hypoxic environment, further complicating the deleterious cell signaling and metabolic dysfunctions associated with obesity [53]. Indeed, it is postulated that a failure in angiogenesis in adipose tissue of individuals with obesity plays a crucial role in the loss of plasticity of this tissue, a process that is amplified by aging [28]. Adipocyte hypertrophy in VAT disrupts and compromises the tissue cytoarchitecture and microenvironment, leading to reduced oxygen availability, inducing chronic hypoxia, which in turn aggravates tissue dysfunction [2, 9, 15, 22] (Fig. 1). Extreme remodeling of the extracellular matrix due to obesity and hypoxia as a triggering factor, induces the development of local fibrosis, which causes adipocytes to lose their plasticity and generate pathological changes [55]. Moreover, hypoxia promotes the production of reactive oxygen species (ROS) [56], a key player in the progression of senescence (Fig. 1). Additionally, hypoxia-induced ROS generation also enhances tissue fibrosis [57, 58], exacerbates insulin resistance [59], and stimulates the release of pro-inflammatory adipokines [60–62]. Exposure to hypoxia or TNF-α induces the release of proinflammatory cytokines and molecules attracting macrophages from preadipocytes, favors chronic inflammation and adipose tissue dysfunction [63]. Collectively, these processes contribute to the pathogenesis of adipose tissue during obesity and metabolic related disorders. Considering the described evidence, it becomes crucial to investigate the multifaceted role of hypoxia-induced senescence in adipose tissue, particularly within the diverse cellular landscape of both SAT and VAT. Given the intricate interplay of various cell types in these tissues, further research is essential to unravel how senescence-induced hypoxia, exacerbated by obesity, contributes pathogenically to each cell type. This investigation is particularly vital to comprehensively understand the mechanisms driving tissue fibrosis, insulin resistance, and the onset of related metabolic and inflammatory diseases. Such insights could help identify novel therapeutic interventions targeting the underlying causes of obesity-related complications.

## The role of autophagy in adipose tissue senescence and disfunction

**Fig. 2:**
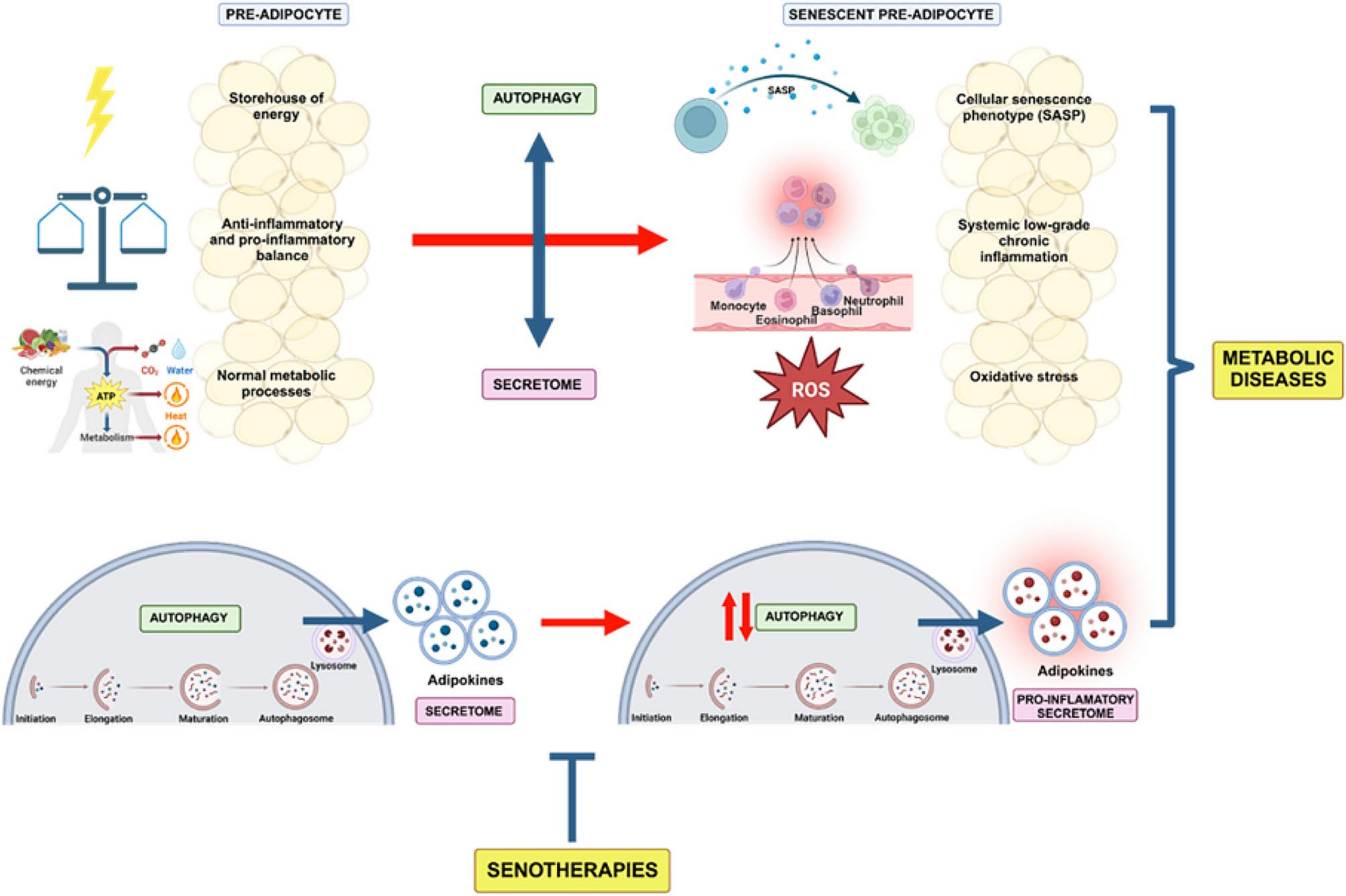
Consequences of senescence in adipose tissue. Healthy adipose tissue plays a key role as an energy source, in the balance between inflammatory and anti-inflammatory pathways through its endocrine function and in several metabolic processes. With aging and the expansion of adipose tissue in obesity, cellular senescence emerges as a significant event in tissue dysfunction. During this process, alterations emerge in different processes fundamental to cellular homeostasis such as autophagy and regulation of the secretome. This triggers cellular phenotype changes towards a senescent phenotype, chronic systemic inflammation and oxidative stress, events which may promote the development of metabolic diseases. The use of natural senolytic or senomorphic agents could decrease the alterations generated by senescence in adipose tissue thus reducing the burden associated with metabolic diseases associated with aging. Created with BioRender.com agreement number ZZ270NXGGE.

Macroautophagy, commonly known as autophagy, is a dynamic and evolutionarily conserved cellular degradation process essential for maintaining cellular homeostasis and enhancing cell survival under stressful conditions [64]. During this process, cytosolic macromolecules and malfunctioning organelles are sequestered and then degraded within lysosomes [53, 55]. Although autophagy primarily serves as a cytoprotective mechanism, its dysregulation can lead to detrimental cellular outcomes, including cellular senescence [65, 66]. In adipose tissue, autophagy plays a crucial role in regulating energy balance and metabolism by overseeing energy storage, facilitating the differentiation from preadipocytes to adipocytes, and managing the turnover of lipid droplets [66, 67]. Interestingly, in the context of obesity, the lipotoxic environment—resulting from the diet and excessive accumulation of adipose tissue—is counterbalanced by increased autophagy in adipose tissue, which acts as a protective mechanism to mitigate disruptions in homeostasis [61]. It is well-recognized that autophagy dysfunction, manifested as either impairment or hyperactivation, contributes to obesity-related disorders [24, 65, 66, 68]. However, the impact of autophagy on the senescence of adipose tissue during obesity is less clear [24, 65, 66]. Numerous studies have shown that autophagy dysregulation triggers senescence in various tissues [69, 70], yet its role in the progression of senescence in adipose tissue remains debated [66]. A significant factor promoting senescence in adipose tissue is ROS. Notably, a high-fat diet and palmitic acid, characteristic of pro-obesogenic diets that negatively affect autophagy [71, 72], increase ROS production [38] and senescence in both adipose tissue and other tissues [69]. Intriguingly, ROS induces autophagy, which in turn serves as a vital cytoprotective mechanism against the oxidative stress associated with obesity [61]. In adipose tissue, the onset of senescence triggered by oxidative stress highlights the potential therapeutic benefits of activating autophagy (Fig. 2). Research using mouse models, specifically those with a deletion of the negative autophagy regulator RUBCN/rubicon [73], demonstrates that increased autophagy can lead to reduced obesity and WAT hypertrophy, when subjected to a pro-obesogenic diet [68] (Fig. 1). Conversely, deleting RUBCN/rubicon in a tissue-specific manner within adipose tissue initiates a fasting-like response, characterized by fat loss, enhanced lipid mobilization, accumulation of lipids in the liver, hepatic steatosis, and a rise in ketone body production [73]. This process ultimately results in the hyperactivation of autophagy and the development of fatty liver disease. It is important to recognize that the response to autophagy manipulation varies significantly across different tissues. Furthermore, these responses are intricately influenced by factors such as an individual’s sex, degree of obesity and body weight [74]. The nuanced role of autophagy, especially its inhibition in adipose tissue, has been identified as a promising target for obesity treatment [61, 75, 76]. This is largely due to the diverse autophagy substrates that accumulate in adipose tissue during obesity [61], suggesting that careful modulation of autophagy could offer a novel approach to mitigating obesity and its associated metabolic derangements (Fig. 2). Future research is imperative to identify agents modulating adipose tissue autophagy, potentially influencing preadipocyte senescence and adipose tissue function.

## Adipose tissue secretome in obesity and senescence

The secretome refers to the complex mixture of bioactive molecules, such as proteins, cytokines, growth factors, and other signaling molecules, that are secreted by cells into their surrounding environment, presenting potential applications as diagnostic biomarkers or therapeutic targets [68]. These secreted factors can have various effects on neighboring or distant cells and tissues and play essential roles in cell-to-cell communication, tissue homeostasis, immune response, and other physiological processes [73]. Adipose tissue’s secretome comprises bioactive adipokines (e.g., leptin, adiponectin), cytokines (e.g., TNF-α, IL-6), growth factors, and other molecules that impact metabolic regulation, immune modulation, insulin sensitivity, and lipid metabolism [74, 75]– [77]. These adipokines exhibit depot- and context-dependent effects, playing roles in physiological or pathological conditions [69] and can be influenced by various factors, such as obesity, inflammation, and nutrient availability [70]. Interactions within the secretome components can significantly impact disease development, offering opportunities for therapeutic and diagnostic interventions. For example, leptin, adiponectin, and resistin stand as extensively studied adipokines, implicated in adipocyte contributions to breast cancer progression [68]. Accumulation of senescent cells in adipose tissue during obesity and aging correlates with changes in the expression of adipogenic factors [71, 72] and altered metabolic and secretory profiles, mounting immune responses which impair their adipogenic response [78]. Notably, the adipose tissue secretome with high levels of different cytokines may originate from senescent preadipocytes, acting as major sources of proinflammatory cytokines [41, 74]. This supports the concept that a senescent secretome can influence neighboring cells and systems, providing insights into the regulation of diverse biological processes (Fig. 2).

## Relationship of adipose tissue secretome, Autophagy, and Senescence

Recent research has increasingly focused on the complex interplay between the adipose tissue secretome and autophagy, exploring their critical roles in cellular maintenance and immune system regulation. Current investigations underscore a reciprocal relationship within adipose tissue, where autophagy dynamically shapes the secretome, influencing the composition and secretion of cytokines and adipokines, among other factors [74, 79, 80]. Dysfunctional autophagy in adipose tissue alters adipokine and cytokine secretion profiles, potentially impacting cell signaling and triggering tissue inflammation, thereby contributing to metabolic disturbances associated with obesity [81, 82]. Furthermore, various components of the secretome of adipose tissue exert regulatory control over autophagy. For instance, adiponectin, known for its insulin-sensitizing and anti-inflammatory properties, enhances autophagy [83], influencing adipose tissue function and different tissues of the body [84]. Insulin-like growth factor 1 (IGF-1) has been identified as a positive regulator of autophagy in adipocytes, contributing to metabolic stability and balance of the immune regulators [85]. In this context, a reciprocal regulatory relationship exists between the NLRP3 inflammasome and autophagy. The NLRP3 inflammasome is a multiprotein complex component of the innate immune system that plays a crucial role in the immune response, particularly in the maturation and release of pro-inflammatory cytokines like IL-1β and IL-18 in response to cellular stress in adipose tissue [86]. Autophagy prevents NLRP3 inflammasome activation by eliminating key elements that trigger inflammation such as damaged organelles, while the inflammasome’s signaling pathways modulate autophagy function [87] (Fig. 2). Galectin-3, another notable protein within the context of the adipose tissue secretome and autophagy, has been identified as a biomarker for metabolic syndrome and obesity, attributed to its elevated levels in the plasma of patients with obesity and type 2 diabetes [88–91]. Intracellularly, Galectin-3 is instrumental in maintaining the homeostasis of damaged lysosomes, facilitating their degradation and repair, which is crucial for cellular health [92] (Fig. 1). Furthermore, research indicates that the deletion of Galectin-3 in adipose tissue impairs its plasticity, adversely affecting insulin sensitivity and glucose homeostasis [93]. While Galectin-3’s involvement in senescence has been documented across various contexts [94, 95], its specific role in adipose tissue senescence remains to be fully elucidated. This gap highlights the necessity for further investigations to establish a comprehensive understanding of how key components of the secretome from different WAT depots contribute to obesity and the development of a senescence phenotype (Fig. 2).

## Regulation of adipose tissue senescence by Senotherapies

To counter the rising prevalence of obesity and its associated health ramifications, it is crucial to implement strategic interventions aimed at preventing or mitigating senescence in adipocytes, thereby reducing the development of metabolic diseases. In response to this need, senotherapies have emerged as a promising approach. Senotherapies are treatments designed to target and manage cellular senescence. These therapies work by either selectively removing senescent cells or modulating their secretome, which can negatively impact tissue function and promote chronic inflammation. By addressing cellular senescence, senotherapies offer a novel way to improve metabolic health and combat obesity-related complications. Senotherapies can be categorized into two primary types: senolytic and senomorphic. Senolytic therapies are designed to selectively eliminate aged cells [96–98], by transiently disrupting anti-apoptotic pathways (SCAPs), which are more prominently expressed in these cells than in their non-senescent counterparts. As a result, senescent cells exhibiting SASP undergo apoptosis [99]. Studies have shown that even modest reductions in the burden of senescent cells can significantly alleviate the associated symptoms [100]. On the other hand, senomorphic therapies aim to mitigate the impact of SASP without inducing cell death [97, 100, 101]. Senomorphic therapies target key signaling pathways—such as p38MAPK, PI3K/Akt, mTOR, JAK/STAT, NF-κB, and STAT3—which are crucial in the regulation of SASP. By attenuating SASP, these therapies neutralize specific inflammatory factors, including IL-1α, IL-8, and IL-6, using targeted antibodies [100, 102]. A combined approach that utilizes both senolytic and senomorphic properties represents a promising strategy to optimize the function of adipose tissue showing signs of senescence. In this context, natural compounds with potent antioxidant properties have become significant, modulating crucial pathways such as mTOR and redox regulation [34, 98]. Various natural and phytochemical products, especially polyphenols, found in fruits, vegetables, seeds, and nuts, exhibit senolytic and senomorphic properties, due to their antioxidant nature [34, 103]– [106]. Indeed, their potent anti-inflammatory actions position them as strong contenders against diseases induced by obesity [107–109]. Polyphenols, which have the potential to regulate oxidative stress, inflammation, cellular senescence, and autophagy, are emerging as key players in the senolytic field [110]. Due to the pleiotropic effects of SASP, natural treatments such as polyphenols could represent a therapeutic strategy, as potential game-changers in preclinical models [111, 112] (Fig. 2). Specific compounds such as rapamycin, resveratrol (RV) and epigallocatechin gallate (EGCG) can act as senomorphics [97]. Others, including quercetin, fisetin, curcumin [97], rosmarinic acid (RA), RV, EGCG, apigenin and olive-derived polyphenols are under scrutiny for their senolytic properties [100, 113, 114] (Table 1).

**Table 1:** Reported effects of different polyphenols on senescence

	Polyphenol	description	Effect on senescence	Ref
1	Rapamycin	Natural macrocyclic lactone produced by the bacterium Streptomyces hygroscopicus, is a well-known mTOR inhibitor.	Decrease cellular senescence and suppress SASP markers, likely through an mTOR pathway	[32, 96]
2	Resveratrol	Natural phenolic compound and has been found in many foods, such as grapes, peanuts, blueberries, and red wine	Has shown life-extending effects in certain study models, particularly in mice fed a high-fat diet.	[101]
			Part of the effects occur through SIRT1 and could be considered as an age-related metabolic modulator	[115]
			Inhibits Ischemia-Induced Myocardial Senescence Signals and NLRP3 Inflammasome Activation	[116]
3	Fisetin	Senolytic-flavonoid polyphenol found in strawberries.	Has demonstrated the potential to restore attenuate age-linked pathologies, and prolong lifespan, even when administered later in life	[117]
			Has been reported as potent senolytic in several animal models as well as in human adipose tissue	[115]
4	Quercetin	Flavonoid frequently found in low amounts as a secondary plant metabolite in fruits and vegetables	Is considered a geroprotective agent for in vitro models of premature aging	[115]
			In combinations with dasatinib, induce apoptosis in senescent cells, ameliorating age-associated alterations in animal models	[97]
			In combinations with dasatinib, reduce senescent cell burden in subcutaneous adipose tissue and alleviate circulating SASP factors in patients with diabetic kidney disease, as demonstrated in a clinical trial	[118]
5	Apigenin	Flavonoid, principally present as glycosylated in vegetables, fruits, herbs, and plant-based beverages	Decrease aging-associated changes due to oxidative stress on vascular endothelial function	[115]
			Suppresses the senescence-associated secretory phenotype and paracrine effects on breast cancer cells	[98]
6	Curcumin	Main bioactive polyphenolic compound that is extracted from the Curcuma longa (Tumeric) rhizomes	Is considered to be a potent senolytic, in part through mTOR inhibition and activation of FoxO	[115]
			Attenuate D-galactose-induced brain senescence in vitro and in vivo	[111]
7	Epigallocatechin gallate	Polyphenolic compound and the major catechin found in green tea	Suppresses premature senescence of preadipocytes by inhibition of PI3K/Akt/mTOR pathway and induces senescent cell death by regulation of Bax/Bcl-2 pathway	[36]

A clinical trial observed heightened SA-β-gal activity in adipose tissue of severely obese individuals, correlating with factors including IGFBP3, PAI1, CCL2, and IL-6. Senolytic treatment demonstrated success in reducing SA-β-gal staining, thereby normalizing these alterations indicating promising directions for application in human health [119].

Notably, chronological aging does not inevitably lead to premature senescence but may be triggered by other stressors, such as an unhealthy lifestyle [120]. For this reason, nutritional senotherapeutics may offer a personalized nutrition approach to improve age-associated outcomes [100] (Fig. 2). Despite the many benefits of polyphenols, the content of polyphenols in foods can vary significantly, making their effects also diverse. The low bioavailability of polyphenols is one of the main limitations commonly described, as it may hinder their biological effect [120, 121]. Thus, the introduction of modifications in the structure and the use of nanotransporters may increase the possibility of using the pharmacological potential of polyphenols to treat various diseases [120]. It is important to understand the nature and distribution of these compounds in the diet to determine their bioavailability, bioaccessibility, digestive stability and intestinal absorption. Since polyphenols can have different chemical structures, it is not possible to immediately quantify their exact content in food. Their beneficial action of polyphenols depends not only on their content in food, but also on other factors such as their stability, microbiota and digestive enzymes [122]. Among the main foods with a known high content of polyphenols are wine, green tea, grapes, red fruits, and coffee [122]. In the case of resveratrol, food samples with high content are grapes, peanuts, among others, and in grapes for example it can contain between 1 and 10 mg per kilogram, content that changes in grape juice or wine depending on the grape variety and extraction procedures [108]. Fisetin, a polyphenol found in fruits, vegetables, teas and Anacardiaceae (Rhus succedanea) plants, has concentrations ranging from 0.1 to 539 µg/g [107]. In this regard, strawberries are one of the food sources with the highest amount of fisetin (160 µg/g) [120]. Studies carried out on wines of different grape varieties showed that red wine contains a higher amount of quercetin being up to 16.21 mg/L [109]. Apigenin is also found in several sources, the most important being wolfberry leaves (547.0 mg/kg) and belimbi fruit (458 mg/kg) [110]. The curcumin content can also vary according to the powder sample analyzed, reporting between 1 and 5.7% curcumin [112]. Green tea leaves have the highest EGCG content and given their low stability due to the environmental conditions of the gastrointestinal tract, such as pH and enzymes, various forms of encapsulation have been postulated with the aim of increasing their biological potential [123].Despite the large number of studies evaluating the effects of polyphenols in humans, few have associated their bioavailability to support their bio-efficacy [124]. This is an important objective for future research. Generally, polyphenols are safe for healthy individuals when consumed as part of a balanced and varied diet. However, in cases of polyphenol supplementation, where the ingested amount can increase significantly, clear negative effects may occur. These include disturbances in iron homeostasis, alterations in the activity of digestive enzymes, changes in the composition of the intestinal microbiota, interactions with various medications, and imbalances in hormonal levels, among others [125]. In conclusion, leveraging the potential of polyphenols and other senolytic or senomorphic agents emerges as a proactive strategy to prevent or reverse adipocyte senescence, thereby restoring adipose tissue function—an effective approach against obesity and its associated health challenges.

## Future directions

As we advance our understanding of adipose tissue dynamics and how changes in it underlie the obesity-related adipose tissue phenotype, future research should aim to dissect the cellular mechanisms that govern the transition of adipocytes into a senescent state. Investigating the interplay between autophagy and the secretome will be crucial for unraveling how these processes collectively contribute to, or help to mitigate, adipose tissue dysfunction. Targeting the pathways leading to senescence and its propagation within adipose tissue may open new therapeutic avenues. Additionally, exploring the role of novel serotherapeutic compounds in modulating adipocyte senescence offers a promising strategy for combating the metabolic complications associated with the development of obesity. Such interventions could redefine approaches to obesity management, ultimately reducing the global burden of related diseases and enhancing the quality of life for affected individuals. Integrating these discoveries with the current progress in clinical and basic science could pave new ways to prevent and treat obesity-related diseases.

## Data Availability

Not applicable.
